# Familial case of TRAPS syndrome in Russia

**DOI:** 10.1186/1546-0096-12-S1-P268

**Published:** 2014-09-17

**Authors:** Evgeny Fedorov, Svetlana Salugina, Maria Soboleva

**Affiliations:** 1 Pediatric, Nasonova Research Institute of Rheumatology, Moscow, Russian Federation; 2 Pediatric, Novosibirsk State Medical University, Novosibirsk, Russian Federation

## Introduction

TRAPS syndrome (tumor necrosis factor (TNF) receptor-1 associated periodic fever syndrome) is a typical representative of periodic auto-inflammatory fevers of monogenic character.We did not find any descriptions of TRAPS syndrome in the Russian population.

## Objectives

To describe the case of TRAPS syndrome in three Russian family generations confirmed by molecular genetic tests.

## Methods

In addition to standard rheumatologist examination, TNFRSF1A gene was partially tested in the patient, his grandmother and parents by direct sequencing with the study of exons 2, 3, 4.

## Results

The patient is a boy of 9 years old of the Russian ethnic origin. The disease manifested at 6 months by fever episodes up to 39.5 °C, lasted for 7-10 days with 4-weeks intervals associated with intensive abdominal pain and increase in acute phase markers (ESR up to 43 mm/h, increase in CRP level, leukocytosis). During the periods between attacks the abovementioned indexes were considerably lower but did not return to normal values. In the follow-up period the patient developed pericarditis episode. At 7 years of age he underwent surgery for peritoneal commissures. In February of 2012 he developed extensive hemorrhagic rash and hematuric nephritis. Glucocorticoid therapy (GT) started in a dose of 0.8 mg/kg had partial effect. The symptoms relapsed with dose reduction. The diagnosis was established in April of 2013 at the age of 9 years. The patient has taken canakinumab since October of 2013 in a dose of 2 mg/kg once every 8 weeks with complete reversal of all symptoms and withdrawal of GT. The patient’s mother (36 years old) has been sick since the age of 4. The disease has been manifested by fever attacks up to 40.0 °C, intensive abdominal pain, periorbital redness and edema. At the age of 14 one of the episodes was evaluated as appendicitis but appendectomy was conducted without effect. Since 16 years she has developed episodes of extensive erythema of an upper limb associated with fever up to 38.0 °C for 3-4 days, transient elbow joint contracture on the side of lesion and intensive abdominal pains. The episodes lasted for 3 weeks and occurred twice a year. Acute phase markers were permanently increased (ESR 58 mm/h, CRP 62 mg/L, leukocytosis up to 12.0 х 10^9^ /L).The patient’s grandmother has clinical presentation similar to daughter’s disease appeared at the age of 14. Mutation of c151C>T (pHis51Tyr) in heterozygous state was found in these three patients. This mutation recorded in Infevers database had been originally described in patients of German and Scottish origin.

## Conclusion

A familial case of genetically confirmed TRAPS syndrome with known mutation originally detected in patients from the Western Europe was found in Russia for the first time. The treatment of the child patient with IL-1β inhibitor lead to complete remission of disease activity and allowed to discontinue glucocorticoid therapy.

## Disclosure of interest

None declared.

